# Identifying environmental drivers of *Aedes aegypti* and *Aedes albopictus* abundance in the Dallas-Fort Worth metroplex using Random Forest modeling

**DOI:** 10.1093/jme/tjaf036

**Published:** 2025-04-10

**Authors:** Nathanial O’Dell, Bethany G Bolling, Nina Dacko, Joseph T Carr, Bethany Hambrick, Luis F Chaves, Joseph R McMillan

**Affiliations:** School of Public Health, University of Washington, Seattle, WA, USA; Department of Mathematics and Statistics, Texas Tech University, Lubbock, TX, USA; Public Health Laboratory Division, Texas Department of State Health Services, Austin, TX, USA; Gulf South Center for Vector Educational Center for Training, Outreach & Resources, Fort Worth, TX, USA; Environmental Health Division, Tarrant County Public Health Department, Tarrant County, TX, USA; Environmental Health Division, Tarrant County Public Health Department, Tarrant County, TX, USA; Department of Environmental and Occupational Health, School of Public Health-Bloomington, and Department of Geography, Indiana University, Bloomington, IN, USA; Department of Biological Sciences, Texas Tech University, Lubbock, TX, USA

**Keywords:** *Aedes aegypti*, *Aedes albopictus*, Random Forest, machine learning, species distribution model, mosquito surveillance

## Abstract

*Aedes aegypti* and *Aedes albopictus* are 2 medically important vectors that have established populations globally. In the United States, *Ae. aegypti* populations declined post-*Ae. albopictus* introduction, though both species now can be readily found throughout the Southern US. Despite overlapping distributions, there are few studies that investigate and compare the drivers of abundance at spatial scales relevant to mosquito control and surveillance districts. To address this limitation, we analyzed longitudinal mosquito surveillance data from the Dallas–Fort Worth metroplex, Texas. Dallas–Fort Worth metroplex is an area of interest due to its rapid population growth, diverse environmental conditions, and prior history with epidemic West Nile virus transmission. We trained a Random Forest model on a subset of *Ae. aegypti* and *Ae. albopictus* data and meteorological and sociodemographic variables from Tarrant and Dallas counties to predict the abundance of both species within the Dallas–Fort Worth metroplex. Additionally, we interpolated predictions to map mosquito abundance at unsampled locations. We found that *Ae. aegypti* abundance was positively correlated with hot and dry conditions within densely populated locations, with mean abundance peaking in the 33rd to the 44th weeks of the year. *Ae. albopictus* abundance was positively correlated with cooler temperatures in higher socio-economic locations with lower human population density, with mean abundance peaking in the 19th to the 32nd weeks of the year. Our results suggest that the diversity of the Dallas-Fort Worth metroplex’s environmental conditions enable *Ae. aegypti* and *Ae. albopictus* to exploit differential niche spaces, which has the potential to influence vector control strategies and disease prevention efforts.

## Introduction

*Aedes albopictus* (Skuse) and *Aedes aegypti* (L.) are competent vectors of Zika, Yellow Fever, and Dengue virus, amongst other diseases (Leta et al. 2018). The spread and domestication of these 2 invasive species has historically varied greatly. *Ae. aegypti* was first reported in North and South America in the 15th century and spread globally through means of global trade out of and through Africa (Powell and Tabachnick 2013). Numerous coordinated efforts in the United States (US) attempted to control *Ae. aegypti* populations with some success, though most were short lived due to a lack of funding (McGregor and Connelly 2021). In contrast, *Ae. albopictus* was first recorded within North America in 1985 in Houston, Texas. Its spread was rapid, with populations being established in 15 states within 3 yr of the initial discovery (Moore and Mitchell 1997). Since their respective introductions into the continental US, both mosquito species are heterogeneously distributed across the United States (Eisen and Moore 2013, Hahn et al. 2016), indicating that they are able to establish themselves along diverse environmental gradients. As such, understanding their spatiotemporal distribution patterns is imperative to various public health initiatives (Smith et al. 2004).

The ecological niches of both species vary. *Ae. albopictus* is primarily a forest edge inhabiting species and has proven capable to adapt to container habitats created inadvertently by humans. Its ability to colonize habitats such as tree holes and small containers has made its distribution difficult to control—especially in the eastern United States (Moore et al. 1988). *Ae. aegypti*, on the other hand, originated as an ecologically opportunistic species that resided primarily in forested locations. However, the species now specializes in living near and biting humans (Rose et al. 2022). Both species oviposit in containers, meaning that they lay eggs in tires, vases, tree holes and other similar objects that can hold water for extended periods of time.

*Ae. albopictus* has shown to have superior larval competitive abilities to the effect of displacing *Ae. aegypti* in the southern US (Lounibos et al. 2002, Kamgang et al. 2018, Muzari et al. 2019). Additionally, evidence of displacement of *Ae. aegypti* by *Ae. albopictus* through means of interspecific mating creating satyr effects has been documented in Florida and China (Tripet 2011, Zhou 2022). However, in recent years *Ae. aegypti* has been detected across the United States, including in the American Southwest, eg, the southern regions of Arizona, New Mexico, and West Texas (Eisen and Moore 2013). In Texas, *Ae. aegypti* was detected in 5 new northwestern Texan counties as recently as 2019 (Greenberg et al. 2019). With global temperatures on the rise, it is likely that both *Aedes* species and thus *Aedes-*borne disease will become more prevalent in Texas (Robert et al. 2020) and as such, it is necessary to conduct longitudinal analyses whenever possible to better understand the complex population dynamics at play between these two medically important species.

Regression models are an important tool that are often used in ecological and epidemiological settings to predict distributions of *Aedes* spp. in both rural and urban locations (Ducheyne et al. 2018, Myer et al. 2020, Rahman et al. 2021). The impact of socio-economic, climatic and landscape variables on predicted adult *Ae. albopictus* and *Ae. aegypti* counts often differ (Rahman et al. 2021), suggesting that while adult populations of these species frequently coexist, their abundance in an area is influenced by distinct factors. Meteorological and landscape variables have long been recognized as being valuable for characterizing *Aedes* spp. distribution patterns (Bidlingmayer 1985); however, in recent years, regression models have increasingly incorporated sociodemographic factors to describe the urban environments where these container-breeding species are typically found (Lin and Wen 2011, Whiteman et al. 2019).

To better investigate the environmental drivers of *Ae. aegypti* and *Ae. albopictus* adult abundance where their distributions are sympatric, we used longitudinal mosquito surveillance data collected from Tarrant and Dallas County, Texas—both located within the Dallas–Fort Worth metroplex (DFWM)—to develop and fine-tune 2 Random Forest (RF) models (Breiman 2001). The objective of our research was to first independently predict the distribution of each species and identify the key climatic, meteorological and sociodemographic factors driving patterns of each species’ abundance. We then interpolated on the predictions from our model to visualize how the distribution of adult female *Ae. aegypti* and *Ae. albopictus* differ throughout the DFWM.

## Methods and Materials

All statistical analyses were done in R V4.3.1 (R Core Team 2023) using a wide range of packages. Spatial data mapping was performed using the WGS84 reference system via the “sf” (Pebesma 2018) and “ggplot2” (Wickham 2016) packages, as well as “leaflet” (Cheng et al. 2023) when finer geographical detail was needed for plots. RF model tuning, training, and validation were done using “ranger” (Wright and Ziegler 2017).

### Study Area

**Fig. 1.**
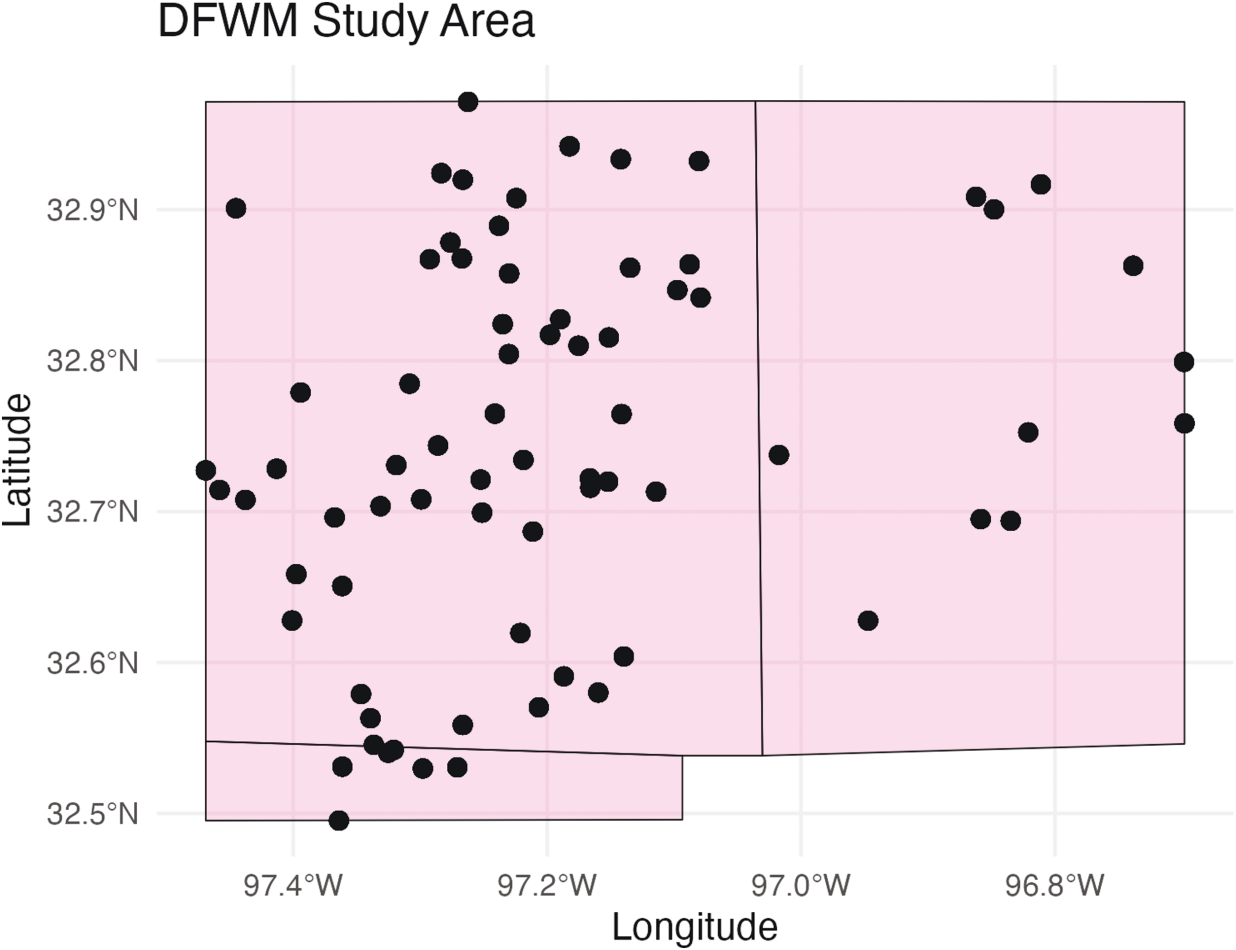
Locations of Gravid traps in the Dallas–Fort Worth metroplex. Trap locations are indicated by circles. Shaded locations indicate the county bounds of our study area.

The DFWM is a rapidly growing collection of cities in North Texas that is amongst the largest metropolitan areas in the United States (Kemper 2005, Lee 2021). Incorporating over 200 cities, the region is home to a diverse range of land cover and socioeconomic conditions as well as population densities (U.S. Census Bureau 2015-2022). The region’s climate is humid subtropical but experiences a wide range of temperatures throughout the year. Summer is the hottest season in the region, with temperatures consistently rising above 32 °C. Precipitation also varies considerably, experiencing anywhere between 50 and 127 cm of rainfall per year (U.S. National Weather Service 2024). Our study area consisted of 14 cities and 3 counties (Fig. 1) where sites were, on average, 24 km apart. Sampling of sites started in 2015 and ended in 2022.

### Data Acquisition

We obtained longitudinal mosquito surveillance data at a weekly temporal scale from Dallas and Tarrant County spanning from 2015 to 2022. The observations in the datasets were collected using a combination of CDC gravid traps, which are typically used in *Culex pipiens* complex surveillance for West Nile Virus (WNV) detection, New Jersey light trap, BG-Sentinel, and CO2 baited CDC light traps, which are all used in *Aedes* spp. surveillance efforts. The data provided by Dallas County represents different submitting agencies. These agencies include city and county health departments, mosquito control districts, military installations, and universities. In Dallas County, mosquitoes are typically collected from May through November by local agencies and then shipped to the state laboratory alive for identification and testing. Factors such as complaints, historical trapping locations, and documented WNV activity drive trap location placement, but these factors are not standardized across agencies. Data from the cities within Tarrant County are collected in a similar manner; however, submissions from local partners are sent to Tarrant County Public Health for identification and testing. Because the surveillance systems conducted by Tarrant and Dallas County public health officials are primarily designed for WNV surveillance, gravid traps were by far the most spatially and temporally replicated between the 2 datasets. While gravid traps are biased against collection of *Aedes* spp., we made the decision to analyze collections in these traps because other trap types were either infrequently set or had a limited surface coverage of the study area.

### Data Cleaning and Preliminary Analysis

**Fig. 2.**
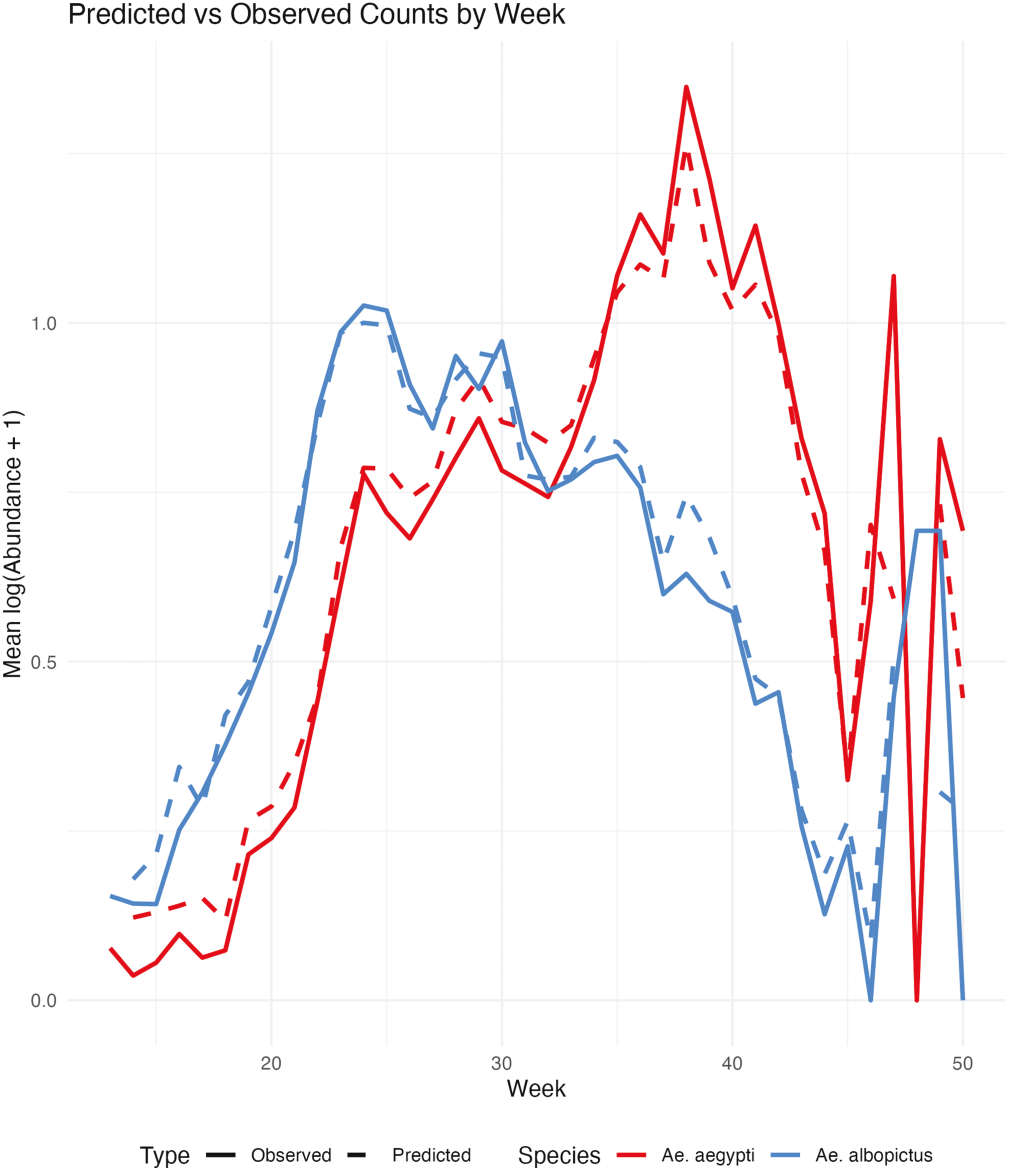
*Ae. aegypti* and *Ae. albopictus* weekly time series. Counts were normalized on a log(abundance + 1) scale in an effort to mitigate the effects of outliers for the purposes of analysis and model building. Dashed lines represent the mean model-predicted values on a weekly temporal scale; solid lines represent mean observed values.

Given the design of these programs, there was considerable statistical noise within each data set that needed to be addressed a priori. First, because the provided data sets only reported *Ae. albopictus* and *Ae. aegypti* counts when a respective species was present, we had to generate true/pseudozero counts. Zero counts for *Ae. aegypti* and/or *Ae. albopictus* were inferred for weeks when *Ae. aegypti* and/or *Ae. albopictus* were not recorded. This adjustment ensured that our data accurately reflected the presence and absence of *Ae. aegypti* and *Ae. albopictus* in each week. After this procedure, *Ae. aegypti* counts were 42.93% zeroes while *Ae. albopictus* counts were 45.93% zeroes (Supplementary Fig. 1), indicating that our counts were zero-inflated (Tu 2002). We then discarded sites that had inconsistent or unusable site coordinates and address names—for instance, some sites had no house number or were otherwise incomplete (eg, addresses with no or multiple zip codes, house number, or street name), while others had multiple or otherwise unusable coordinates due to formatting errors (eg, “32 49.007E”, “6 48.052E” as a latitude measurement, or “32 52 53.0336N” without an accompanying easting measurement) associated with them. Additionally, sites that were not sampled in most years (ie, 5 yr) were removed. This was done because climate variables—such as temperature, humidity, and wind speed—can fluctuate significantly from year to year, influencing mosquito populations. By excluding sites that were not sampled most years we can ensure that the temporal variation in environmental conditions is adequately captured and that sites with inadequate data would not be weighted against sites that had an abundance of data. Doing this left us with 73 sites (out of 375 potential sites) and 10,006 observations. For analysis purposes, we transformed trap counts on a log(*abundance* + 1) scale (Fig. 2).

### Covariate Extraction

We obtained and appended meteorological, land cover and usage, and socioeconomic data from a wide variety of sources (Supplementary Table 1). Daily weather values (eg, temperature means, minimums, maximums, etc.) at a 4 km resolution were extracted using the Oregon PRISM project R package (Hart and Bell, 2015) at a 100 m buffer from each trap location, with data extracted being a coverage-weighted mean that were then averaged at a weekly scale, and cropped to our study area’s spatial extent before being extracted to the 100 m buffer. The 100 m buffer was chosen as an estimate of relevant habitats for *Aedes* spp. based on flight distances (Moore and Brown 2022). Other meteorological data (eg, wind speed, cloud cover, etc.) and its effect on both hindering and aiding *Aedes* spp. sampling has been well documented (Marcantonio et al. 2019, Yin et al. 2019, Myer et al. 2020, Adeleke et al. 2022). Accordingly, further meteorological variables were extracted at a 9 km resolution in a similar manner to the PRISM variables using the “Open-Meteo” package (Pisel 2023), though point data (eg, longitude and latitude coordinates) were used due to package limitations (ie, covariate values are from the cell in which a given site coordinate fell). The data from this Application Programming Interface (API) came in the form of both daily and hourly averages; in both cases, observations were averaged to a weekly temporal scale. For both weather and other meteorological variables, lags up to 3 wk were also created (da Cruz Ferreira et al. 2017).

Gridded Population of the World (GPW) v4 data (Center For International Earth Science Information Network-CIESIN-Columbia University 2018) for years consistent with our sampling window were used to extract population density and percent impervious surface statistics at a 5 km resolution and 100 m buffer. Other land usage statistics were extracted using the “osmdata” package (Padgham et al. 2017). Structure density metrics were defined by creating a 100, 250, and 500 m buffer around trap locations and calculating how much of that buffer was occupied by a respective structure’s polygon (eg, buildings) or line (eg, roads). These distances were meant to reflect the widely varying reported flight range of both species (Honorio et al. 2003, Marini et al. 2019, Moore and Brown 2022), as well as for the purposes of evaluating how different spatial scales effected prediction capabilities. The selected structure types (eg, park, public services) were used to further represent the socioeconomic conditions of sites.

Sociodemographic statistics (Supplementary Table 1) were extracted to site locations using the American Community Survey (ACS) 5-Year Estimates at the census tract level using the “tidycensus” package (Walker and Herman 2023). These estimates were then clipped to our observation data’s spatial extent in the form of a raster with mean values for each variable. These raster values were then extracted to site locations. We chose census variables to extract based off what we believed would best indicate whether a site’s region was urban and high-income or not, and included variables such as median home income, median housing age, and percentage of population below the poverty line.

### Model Calibration

RF is a decision-tree model that has the capability to serve classification or regression purposes (Breiman 2001). This is done by growing a binary tree where at each node in the tree we apply a test to one of the inputs. Depending on the outcome, we go to either the left or right of the sub-branch of the tree until we reach a leaf node, where a prediction is made. This prediction aggregates all training data which reach that leaf. When considering bagging, these decision trees are built on bootstrapped training samples. Bootstraps are conducted by taking multiple samples from a single data set. We then train our model on the *b*th bootstrapped training set to get,


$$\begin{array}{l} {\hat{f}}_{bag}\left(x\right)=\;\frac{1}{B}\underset{b=1}{\overset{B}{\sum }}\;{\hat{f}}^{*b}\left(x\right) \end{array}$$

where *B* is the number of different bootstrapped training sets, and ${\hat{f}}^{*b}\left(x\right)$ is the *b*th prediction (James et al. 2013).

For RFs, each time a split in a tree is considered, a random sample of *m* predictors is chosen as split candidates from the full set of *p* predictors. Thus, at each split in a given tree the majority of predictors are not considered.

RF enjoy good predictive performance with minimal tuning, though tuning of hyper-parameters—parameters not found directly in the training data such as tree size or the *m* number of *p* predictors to consider at each split—can still lead to marginal increases in prediction accuracy (Boehmke and Greenwell 2019). Additionally, unlike many machine learning methods, RF is nonparametric and thus well suited for what might be considered small or otherwise low-dimensional datasets that have non-normal distributions (Ali et al. 2012). As such, RF has been utilized in a wide range of epidemiological studies to predict presence/absence and species counts of mosquitoes with results that frequently outperform other machine learning methods such as generalized linear models and artificial neural networks (ANN) (Cianci et al. 2015, Zhao et al. 2020, Rhodes et al. 2022, 2023).

We prepared our data for model calibration by removing variables that had near zero variance—and thus low predictive power (Boehmke and Greenwell 2019)—using the “caret” package (Kuhn and Max 2008). The respective datasets were then randomly separated into a 75/25 training and testing data split. Prior to model training, reverse feature elimination methodology was used to evaluate subsets of 25%, 50%, 75%, and 100% of the variables. The subset incorporating all 48 variables yielded the highest predictive performance for both models. We then trained our model without tuning hyperparameters to determine the baseline performance (Supplementary Table 2). Afterwards, we utilized a full Cartesian grid search to find the *mtry* (number of variables to split at each node), *minimum node size* (minimum node size to split at), and *sample fraction* (fraction of observations to sample) parameters that yielded the best RMSE and predictive *R*^2^ results (Boehmke and Greenwell 2019). In both the default and tuned models, 1,000 trees were used. Variable importance metrics were then run on the tuned models and represented the mean increase in a model’s RMSE after permuting a feature (Molnar 2020). In this importance method, variables are considered more “important” if shuffling its values increases the model error.

After validating results using the testing data and running model diagnostics, a Moran’s I test was conducted on both models’ residuals to determine if spatial autocorrelation effects were present. In the case of spatial autocorrelation being present, we re-calibrated our RFs forcing site coordinates to be included in all versions of our model(s).

After fitting semivariograms for both species to determine areas of low confidence to mask in interpolations (Supplementary Fig. 6), RF predictions were interpolated on using inverse distance weighting (IDW) interpolation on a 5 × 5 km grid to map the potential distributions of *Ae. aegypti* and *Ae. albopictus* in unsampled locations of DFWM (Mwangungulu et al. 2016). This approach allowed us to extend our RF model predictions across the entire study area—especially valuable for visualization given the sparse and temporally variable sampling—while incorporating spatial dependencies, as a covariate. A key assumption to IDW interpolation is that sites that are closer together can be expected to be more similar than those that are further apart. As such, each sampled point has an influence on the surrounding unsampled area that diminishes as you move further away. IDW interpolation can be computed as:


$$\begin{array}{l} \hat{Z}\left(s_{0}\right)=\frac{\left(\sum_{i=1}^{n}Z(s_{i})\times \left(\frac{1}{d_{i}^{\beta }}\right)\right)}{\left(\sum_{i=1}^{n}\left(\frac{1}{d_{i}^{\beta }}\right)\right)}=\sum_{i=1}^{n}Z(s_{i})\times w_{i}, \end{array}$$

where $\hat{Z}\left(s_{0}\right)$ is the predicted value at $s_{0}$, *n* is the number of sampled locations, $\hat{Z}\left(s_{i}\right)$ is the value at location *s*_*i*_, and *d*_*i*_ is the distance between location *s*_*i*_ and *s*_0_ where we want to predict. The weight is given by $w_{i}={}^{\left(1/d_{i}^{\beta }\right)}/{}_{\left(\sum_{i=1}^{n}\left(1/d_{i}^{\beta }\right)\right)}$, where *β* is the distance power that determines the degree to which nearer locations are preferred over more distant locations (Moraga 2019). For our purposes we used the default distance power of *β* *=* 2.

We used a 5-fold cross validation to evaluate the performance of the interpolation. We define *k*-fold cross validation as:


$$\begin{array}{l} RMSE\;=\;{\left(\frac{1}{n_{test}}\sum_{i=1}^{n_{test}}{\left(y_{i}^{test}-(\hat{y}{)}_{i}^{test}\right)}^{2}\right)}^{\frac{1}{\text{2}}}, \end{array}$$

where $y_{i}^{test}$ and ${\left(\hat{y}\right)}_{i}^{test}$ are the observed and predicted values of observation *i* in the test set, and $n_{test}$ is the number of observations in the test set.

## Results

### Summary Statistics

Of our 10,006 observations, 17.71% had no collections of either *Ae. aegypti* or *Ae. albopictus*, and 28.84% had collections of both. In 53.45% of collections *Ae. aegypti* and *Ae. albopictus* were not recorded within the same week, indicating that co-occurrence within weekly trap collections was uncommon (Supplementary Table 3). However, across all years, at least one individual of each species was found in the same site. *Ae. aegypti,* on average, reached peak abundance later in a season than *Ae. albopictus* and usually had higher mean abundance across all sites (Supplementary Table 4), with each species’ peak usually occurring around weeks 34 and 28, respectively (Fig. 2). Raw summed species counts were 23,856 and 19,330 for *Ae. aegypti* and *Ae. albopictus*, respectively. After transforming weekly counts on a log(abundance + 1) scale, summed counts across all years were 7,774 and 6,968, respectively. Zero-excluded medians of transformed counts were 1.099 and 0.6931, respectively.

The maximum temperature recorded in our study area was 43.362 °C and occurred during July 2018. In weeks in which max temperatures exceeded 32 and 35 °C—temperatures at which blood feeding activity is impacted significantly for *Ae. albopictus* and *Ae. aegypti*, respectively (Reinhold et al. 2018, Costanzo and Occhino 2023)—transformed mean *Ae. aegypti* counts were 0.8507 (SD: 0.8457) and 0.8383 (SD: 0.7947), and mean *Ae. albopictus* counts were 0.7783 (SD: 0.8125) and 0.8235 (SD: 0.8014).

### Aedes aegypti Abundance Model

Final model performance with location as a predictor had an *R*^2^ of 0.3752 and an RMSE of 0.649 (95% CI: 0.632 to 0.667) (Supplementary Table 2). Mean log predicted abundance (per site) ranged from 0.309 to 1.254 (SD: 0.2031), with an overall predicted abundance range of 0.0193 to 2.910 (SD: 0.4683).

Permutation variable importance metrics revealed that the top 5 variables (Supplementary Fig. 2) included mean wind speed at lag 1 (week) (Importance: 0.0817, SD: 0.0017), maximum temperature at lag 2 (0.0704, SD: 0.0014), minimum temperature at lag 2 (0.0635, SD: 0.0006), mean dew point at lag 2 (0.0606, SD: 0.0009), and road density within a 500 m buffer (0.0564, SD: 0.0010). Sociodemographic factors, namely mean home value and poverty percentage—measured by comparing a family’s income against a minimum amount to cover basic needs (U.S. Census Bureau 2015-2022)—were amongst the lowest in importance. Meteorological factors varied in importance, with variables recorded during the week of collection such as cloud cover percentage, minimum and maximum temperature and mean wind speed being within the top 15 variables for the model, while others such as precipitation, vapor pressure and atmospheric pressure were outside of the top 26 (50% of all variables used in our model).

Diagnostic statistical analyses were run on model residuals to test for autocorrelation effects and the assumption of independence. A Moran’s I was run on model residuals using a distance matrix and autocorrelation effects were found (*P* = 0.0115; Moran’s *I* = −0.0020). To account for this, we re-ran our models forcing them to include latitude and longitude values at all optimization steps. Additionally, we plotted error size as a function of predicted values (Supplementary Fig. 3). The line of best fit indicated that the independence assumption was not violated. Finally, we tested for temporal correlation by examining lag effects (Supplementary Fig. 4) using the *pacf* function in the “tseries” package (Trapletti and Hornik 2024).

### Aedes albopictus Abundance Model

Tuned model performance had an *R*^2^ of 0.3059 and an RMSE of 0.652 (95% CI: 0.634 to 0.671) (Supplementary Table 2). Mean predicted abundance (per site) ranged from 0.407 to 1.445 (SD: 0.2397) with an overall predicted abundance range of 0.0130 to 2.744 (SD: 0.4024).

Permutation variable importance metrics revealed that the top 5 variables (Supplementary Fig. 2) included mean dew point (0.1333, SD: 0.0012), minimum temperature at lag 1 (0.1116, SD: 0.0012), mean temperature (0.1067, SD: 0.0009), mean dew point at lag 1 (0.1059, SD: 0.0013), and relative humidity at lag 2 (0.1050, SD: 0.0009). Unlike the *Ae. aegypti* model, sociodemographic factors, namely mean home value and mean income, were within the top 15 important variables. Climate and meteorological factors again varied in importance, with cloud cover at lag 1, mean wind speed at lag 1, and precipitation being within the bottom 5 important variables to our model.

Similar to the *Ae. aegypti* model, diagnosis analyses of the *Ae. albopictus* model was conducted. A Moran’s *I* was computed, and no spatial autocorrelation effects were found (*P* = 0.2343484). Assumptions of independence within our model were also not found to be violated (Supplementary Fig. 3).

### Model Comparisons

**Fig. 3.**
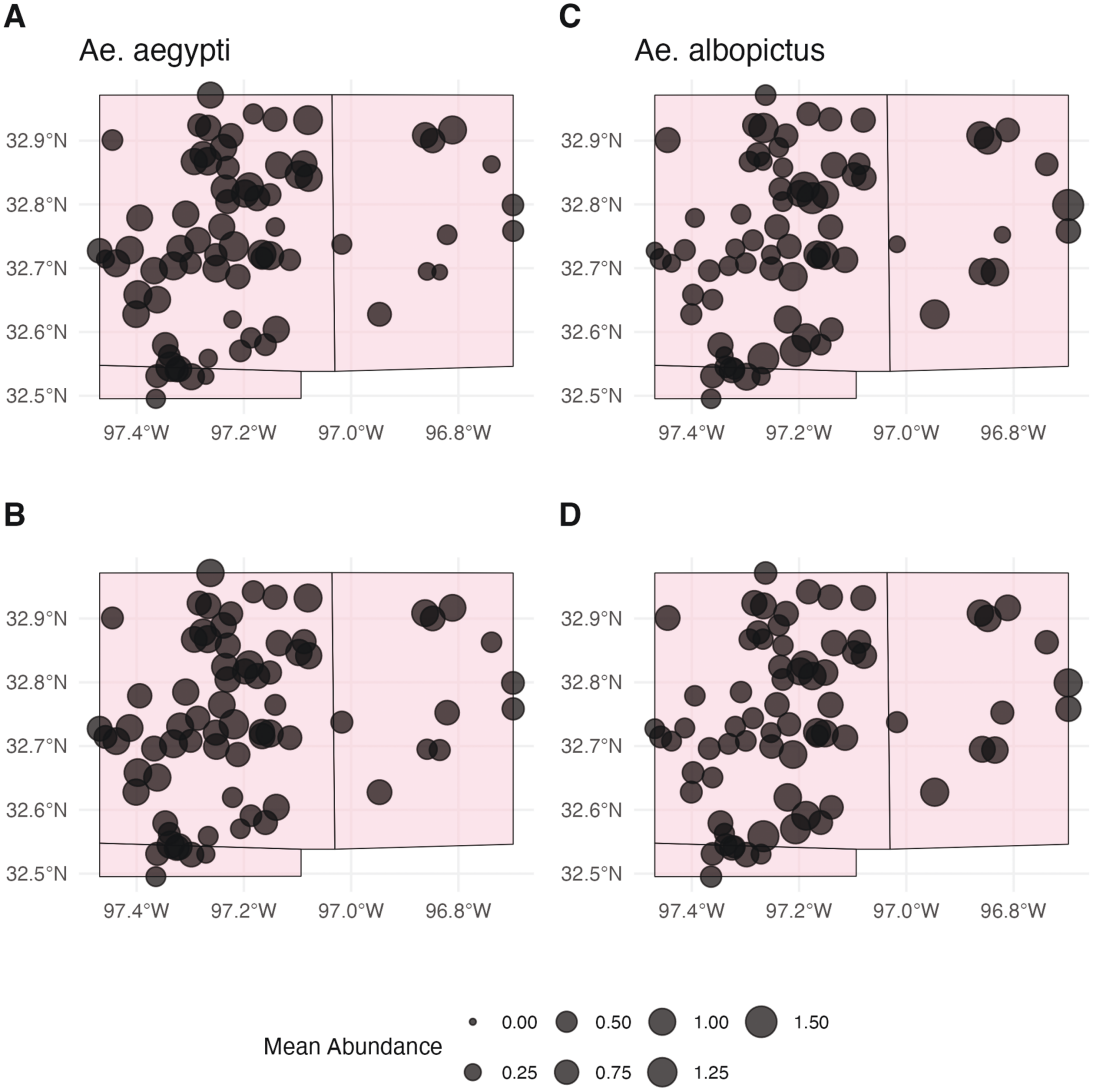
Spatial distribution of mean counts by species. Plots (A) and (B) represent mean observed and predicted counts for *Ae. aegypti*, respectively. Plots (C) and (D) represent mean observed and predicted counts for *Ae. albopictus*, respectively.

**Fig. 4.**
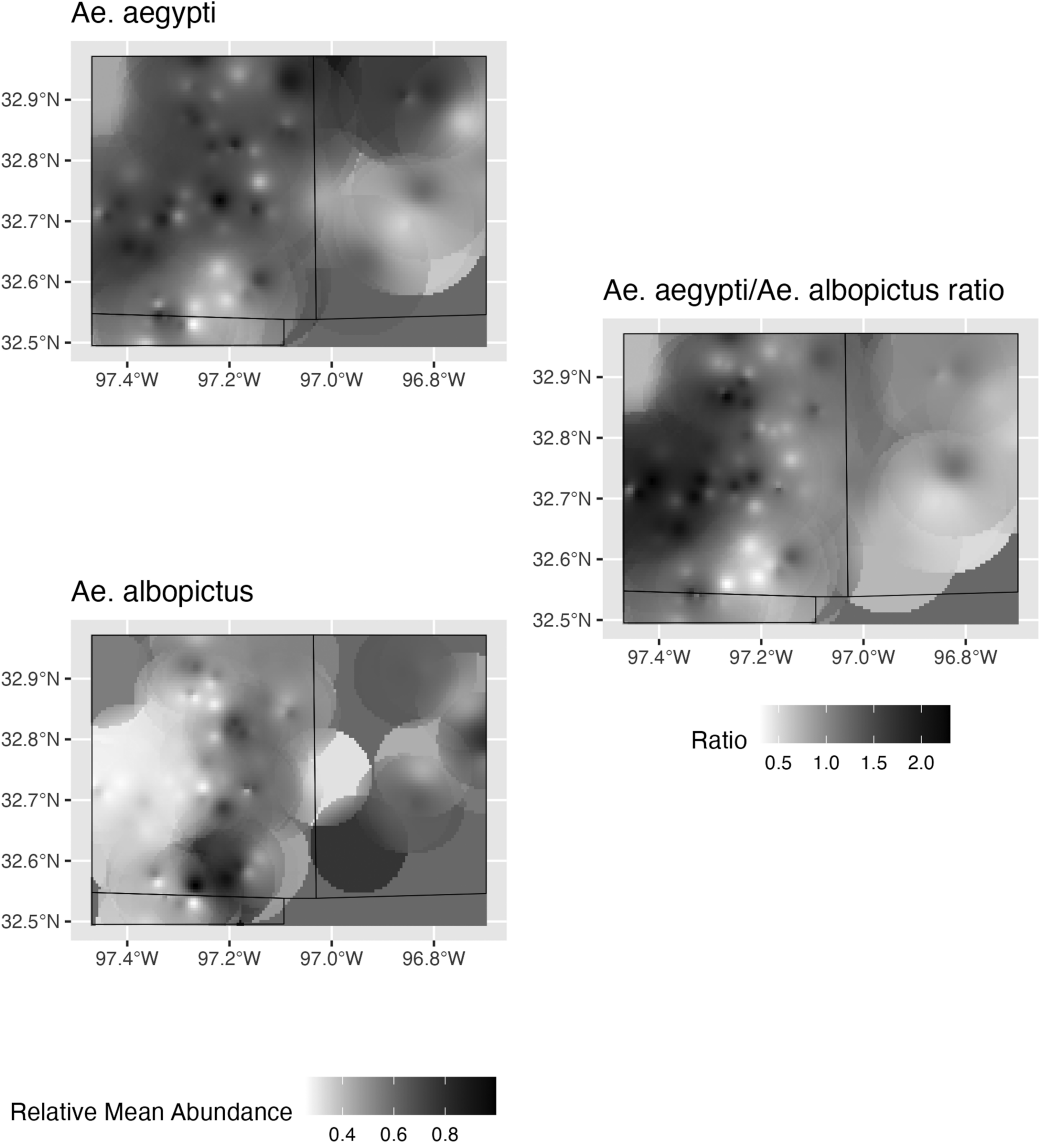
Visualization of potential distribution for *Aedes* spp. counts across the Dallas–Fort Worth metroplex. Values were interpolated by IDW. Values are expressed as mean abundance with respect to species’ relative maximums. Values less than 1 in the third panel indicate *Ae. albopictus* dominance, while greater than 1 indicates *Ae. aegypti*.

The maps in Fig. 3 show the training data abundance distribution and the model outputs. When plotting model predictions at the weekly temporal scale, *Ae. aegypti* generally had higher counts than *Ae. albopictus* in the later weeks of our observation window (weeks 33 to 50) and had the highest number of predicted counts in week 38. *Ae. albopictus* had higher counts earlier in the year (weeks 13 to 22), with its highest number of predicted counts occurring in week 25 (see Fig. 2). Spatially, models predicted lower mean relative counts for *Ae. albopictus* than *Ae. aegypti* in the south-western region of Tarrant County, with generally low mean relative counts for both species in Dallas County. Spatially, areas of high relative abundance (calculated as predicted abundance at a given site divided by maximum predicted abundance across the data set) for *Ae. aegypti* were determined to be areas of lower relative abundance for *Ae. albopictus* (Fig. 4). The *Ae. aegypti* IDW model had an RMSE of 0.1449 (95% CI: 0.1250 to 0.1730) while the *Ae. albopictus* model had an RMSE of 0.1445 (95% CI: 0.1240 -to 0.1720). The relative *Ae. aegypti* abundance model had an RMSE of 0.4096 (95% CI: 0.3530 to 0.4890). Masking for all interpolations is a function of the semivariogram of a respective species’ model and begins at 13 km for *Ae. aegypti* and 9 km for *Ae. albopictus*.

Partial dependence plots, which seek to demonstrate the effect a variable has on a variable of interest—in this case, *Ae.* spp abundance—shown in Supplementary Fig. 5 further illustrate the different relationship that each species has to variables in the model. *Ae. aegypti* counts, for example, have a positive correlation with the percentage of residents in poverty, while the correlation of that same variable with *Ae. albopictus* is negative. The inverse is seen in mean dew point, with *Ae. aegypti* having a negative correlation and *Ae. albopictus* having a positive correlation. Other variables such as minimum and maximum temperature, mean wind speed, and mean income have similar relationships with both species’ counts, though the exact nature of them differ slightly.

## Discussion

Although *Ae. aegypti* and *Ae. albopictus* are broadly distributed throughout the United States, there is a lack of understanding of their distribution outside of subtropical and coastal locations where the 2 species coexist. Information obtained through such studies is critical to address given the impact of climate change and the continuous variation of land use (especially in rapidly expanding locations such as DFWM) on medically important mosquitoes’ distribution patterns (Norris 2004).

For both species, abundance was positively related to maximum temperatures. We found no evidence of an upper temperature limit within model predictions. However, *Ae. aegypti* abundance showed a positive response at a lower temperature threshold than *Ae. albopictus*, suggesting that while the general trends are similar for both species, their specific responses to temperature vary. The negative relationship between mean dew point—which is a key indicator of humidity—and *Ae. aegypti* abundance suggests that this species can effectively distribute across DFWM in drier conditions. Coexistence of both species because of high *Ae. aegypti* egg survivorship during dry seasons within urban landscapes like DFWM have been found and examined in past studies (Reiskind and Lounibos 2013). This could potentially explain why both predicted and observed weeks of peak abundance for *Ae. aegypti* are in the autumn periods; typically, the Dallas-Fort Worth region experiences drier conditions during the later months than those in the summer. For *Ae. albopictus,* lower dew points do not appear to have a negative correlation with their abundance. This is to be expected, as *Ae. albopictus* has been shown to be more robust to a wider range of humidity-related metrics (Reiskind and Lounibos 2013).

Within an independent variable correlation matrix, mean dew point had a significant, positive correlation to minimum and maximum temperatures (*P* < 0.001). The importance of dew point and minimum/maximum temperatures to both species’ abundance might be a key insight into *Ae. aegypti*’s distribution in the region, as DFWM’s hot and seasonally dry conditions are favorable to the species’ abundance patterns. Understanding and further examining this insight is especially important due to the thermal biology of the diseases they transmit. Both dengue and Zika have high thermal optima (~29 °C), suggesting that as temperatures continue to increase and conditions for *Ae. aegypti* and *Ae. albopictus* become more suitable, locations are at a higher risk of not only higher mosquito abundance but also higher rates of disease transmission (Mordecai 2019). Further studies in urban locations with similar socioeconomic and climatic conditions to DFWM are needed to better understand and test this assumption. Although it might be expected that precipitation would be a more important predictor in the models due to its correlation to humidity, DFWM did not experience high rainfall in our observation window (the average weekly rainfall for our sites was just 2.908 cm). The lack of importance of precipitation may also be related to the fact that both species are container breeders, and as such both rain and tap (ie, piped) water can play an important role in creating suitable breeding conditions in urban areas (Barrera et al. 1993, Hayden et al. 2010, Brown et al. 2023).

Mean income of households showed a distinct and varying influence on species-specific counts. *Ae. aegypti* abundance’s relationship to mean income was less sensitive to changes in income than its counterpart; that is, slight fluctuations in income levels did not significantly impact predicted *Ae. aegypti* counts. This effect has been seen in past studies examining *Ae. aegypti* abundance in Arizona (Coalson et al. 2023). The relationship between abundance and poverty percentage, however, is negative only for *Ae. albopictus*. While the association between *Ae. aegypti* and poverty percentage was of low variable importance, rising maximum temperatures in Dallas, Texas because of an increase in impervious surfaces, deforestation, and thus urbanization have been shown to be an indicator of lower mean income and higher unemployment rates (Moss and Kar 2020). We believe it is possible that the high importance of weather-related variables within our model points to weather being the strongest signal capturing the environmental context of variability in socio-economic data.

Population density, which has a slightly negative correlation to mean income (*P* < 0.001), and presence of impervious surfaces—which we would expect to be higher in urban locations and is within the top 15 variables of both species—are both useful for describing the level of development of a site’s surrounding location. Both metrics have a negative effect on *Ae. albopictus* abundance but a positive effect on *Ae. aegypti*. Additionally, we believe that the usage of aerial photographs by vector control specialists and surveillance officials can serve to further validate these findings, providing additional context for the relationship between urban development and mosquito distribution to the effect of identifying cold and hotspots of *Aedes* spp. distribution.

Model results point to *Ae. aegypti* thriving in densely populated, urban areas with high temperatures and low wind speeds, such as those that describe the DFWM. The region has a history of extreme conditions (Foss and Ko 2019) such as a record-breaking heatwave in 2011 and a population growth rate that consistently exceeds national averages (Lee 2021). Our model suggests that *Ae. aegypti* can distribute across a wider region due to its ability to exploit highly variable climates and socioeconomic conditions. Additionally, because of the diverse landscape of DFWM, *Ae. aegypti*’s apparent ability to inhabit the region successfully has not led to a displacement of adult *Ae. albopictus* outside of the effect of seasonal trends. Investigations into the dynamics of these 2 species in the DFWM region during life stages more susceptible to competitive displacement, namely the larval stage, are needed to confirm or deny this assumption (Lounibos et al. 2002). *Ae. albopictus*, which peaks in abundance in the late spring exhibits relationships with environmental and sociodemographic variables that frequently contrast with those of *Ae. aegypti*. This is important to explore further due to the implication of increased risk of vector-borne disease due to the contrasting seasonality of both *Ae. aegypti* and *Ae. albopictus*. *Ae. aegypti*’s higher abundance in the late summer/autumn weeks puts human populations at higher risk of disease transmission. As such, understanding these seasonal variations is crucial for assessing how the comparative abundance of these species may influence overall transmission trends.

One important way our modeling approach differs from other investigations of *Ae. aegypti/Ae. albopictus,* is our use of nuanced meteorological variables such as wind speed and atmospheric pressure. For adult mosquitoes, wind speeds can both facilitate and hinder mosquito flight which may play a role in spatiotemporal distribution patterns (Hribar et al. 2010). Additionally, atmospheric pressure has been shown to be negatively correlated to dengue transmission rates (Sang et al. 2014). We believe our models have shown the potential usefulness of including such meteorological variables as independent variables in future modeling efforts. Independently, sociodemographic and land cover variables only marginally improved model performance. Rather it is the implicated interactions between all variables that improve accuracy. Further analysis should be conducted to better understand their potential usefulness for *Aedes* spp. distribution modeling purposes.

Limitations of our study include uneven geographical trap placement after excluding sites based off our described criteria; of our 10,006 observations, 81% were within Tarrant County. This affected not only the scope of our conclusions, but also likely introduced edge-effects into mapping efforts. Additionally, sites were sampled inconsistently, which led to many potential sites being discarded in the initial cleaning phase. Future efforts should aim to examine a larger, more evenly distributed pool of sites. In attempting to use observations recorded using gravid traps, which were not originally created for *Aedes* spp. trapping, a potential source of bias was introduced to our regression analysis. WNV surveillance systems such as those that describe our data are not designed to investigate *Aedes* spp. abundance systems. However, our results can better inform DFWM public health officials where to focus future surveillance efforts more directed towards these 2 species.

## Conclusions

Our research investigated differences in adult distribution patterns of *Ae. aegypti* and *Ae. albopictus* to identify the environmental factors associated with *Ae. aegypti*’s re-emergence in the southern US and its continued co-existence with an established competitor (Braks et al. 2004). We found that *Ae. aegypti* was more abundant during hotter, drier conditions in densely populated locations of low socioeconomic status in the DFWM. *Ae. albopictus*, on the other hand, was able to distribute effectively even in lower minimum temperatures and dew points and was found in locations with lower population density and higher socioeconomic status. These results suggest that climate and meteorological extremes combined with the incredibly rapid deforestation and thus increasing urbanization of Dallas-Fort Worth is creating greater opportunities for *Ae. aegypti* and *Ae albopictus* to not only co-exist across spatial extents but also presents opportunities to refine niche spaces. In response to the increasing risk of *Aedes-*borne disease, we suggest that north and west Texas public health officials move rapidly to adopt the appropriate surveillance systems to better understand and prevent the growing risk of *Aedes*-borne diseases in the region. Further analysis should be done to the effect of conducting mosquito surveillance data analysis on *Aedes* spp. counts obtained from autocidal gravid ovitrap, BG Sentinel, and/or CDC light traps. Doing so could serve to reinforce the findings here while removing biases we may have introduced with our choice of data.

## Supplementary material

Supplementary material is available at *Journal of Medical Entomology* online.

tjaf036_suppl_Supplementary_Material

## Data Availability

All underlying mosquito surveillance data are available upon reasonable request from either BH or BB.
