# Supplementary material for: Identifying environmental drivers of Aedes aegypti and Aedes albopictus abundance in the Dallas-Fort Worth metroplex using Random Forest modeling
Source: J Med Entomol. 2025 Apr 10;62(4):789–99. doi: 10.1093/jme/tjaf036 (PMC12271727; doi:10.1093/jme/tjaf036)
Supplement: tjaf036_suppl_Supplementary_Material [file tjaf036_suppl_supplementary_material.docx]

**Supplemental Figure 1**

**
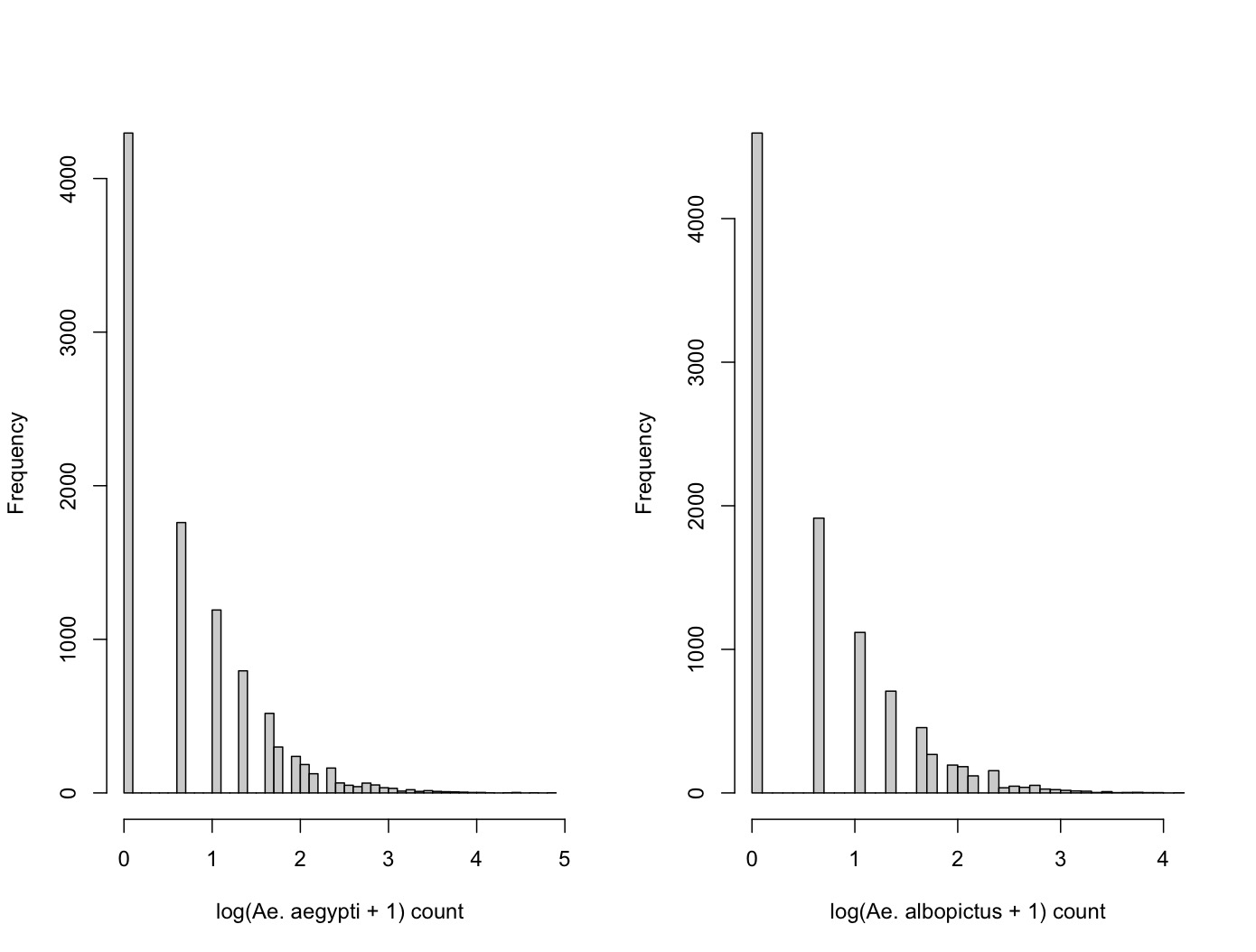
**

**Supplemental Figure 1.** *Ae. aegypti* and *Ae. albopictus* count distributions. The counts exhibited a zero-inflated distribution, with a median of .6931 for both species and a standard deviation of 0.8392722 and 0.7851011 for *Ae. aegypti* and *Ae. albopictus*, respectively.

**Supplemental Figure 2**


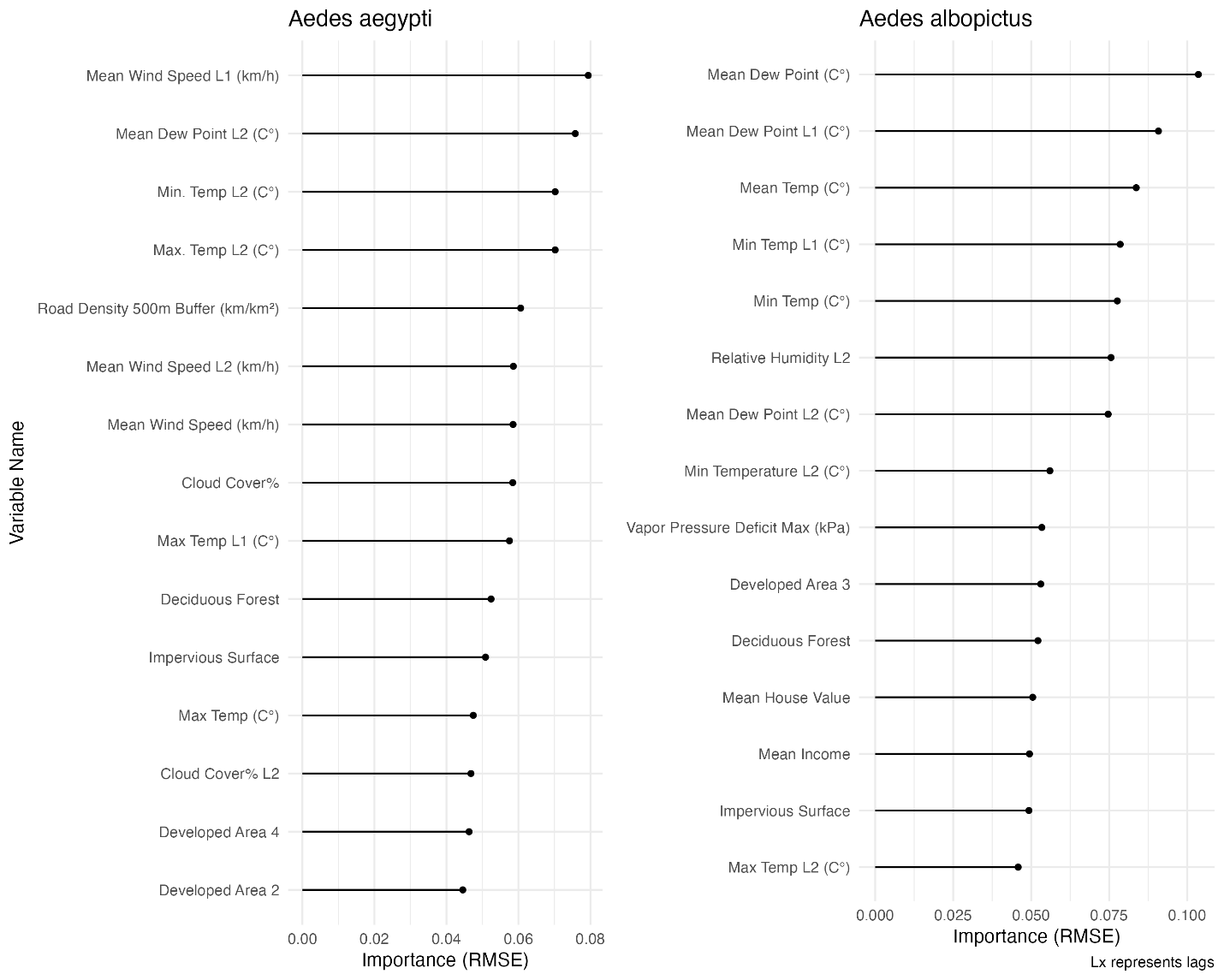


**Supplemental Figure 2.** Permutation variable importance plots of both models. The left panel plots the fifteen most impactful variables on the Ae. albopictus model, and the right panel plots the Ae. aegypti model’s most impactful variables.

**Supplemental Figure 3**


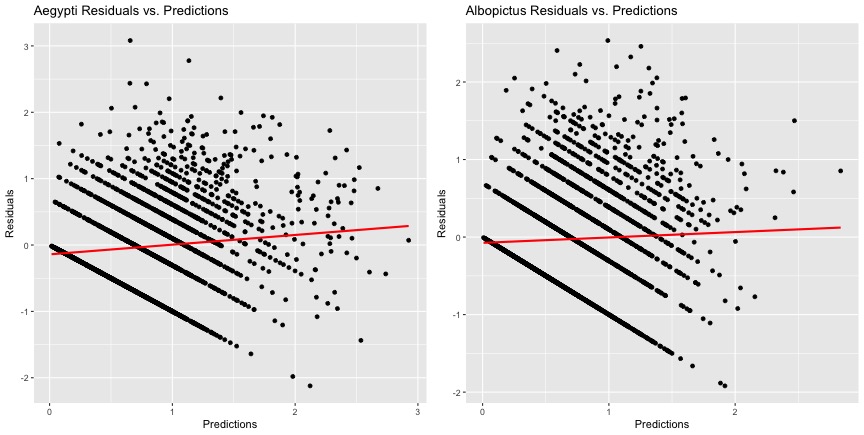


**Supplemental Figure 3.** Residual analysis for both species’ abundance models, where error size is a function of predictions. The line of best fit (red line) indicates that the relationship between predicted values and residuals was non-linear.

**Supplemental Figure 4**
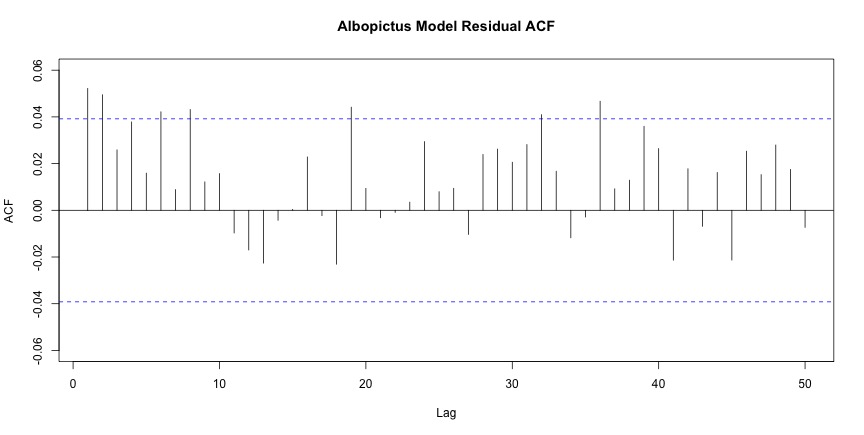

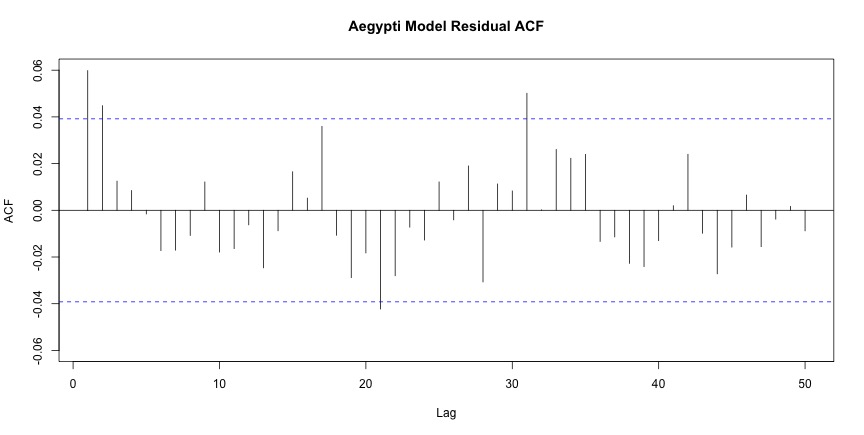


**Supplemental Figure 4.** Autocorrelation function (ACF) plot of model residuals for predicted weekly species abundance. Lags are on a weekly time scale. The dashed line indicates the 95% confidence interval threshold. For a time-series to be considered white noise (i.e. not temporally autocorrelated) we would expect 95% of lags to lie beneath the significance threshold. About 92% of *Ae. albopictus* residuals are beneath the threshold and 94% of *Ae. aegypti* residuals are beneath the threshold.

**Supplemental Figure 5**


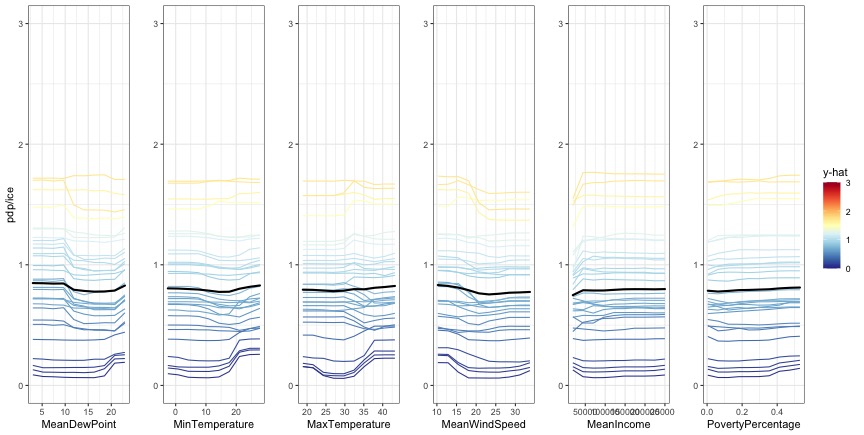

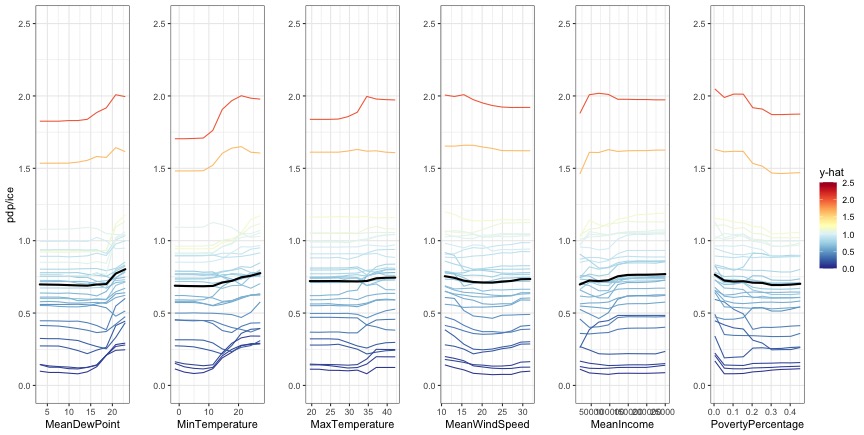
**Supplemental Figure 5**. Partial dependence plots (PDP) of *Ae. aegypti* and *Ae. albopictus*, respectively. Individually colored lines illustrate the dependence of our prediction on a feature for each sample. The solid black line represents the average of all of these values.

**Supplemental Figure 6**


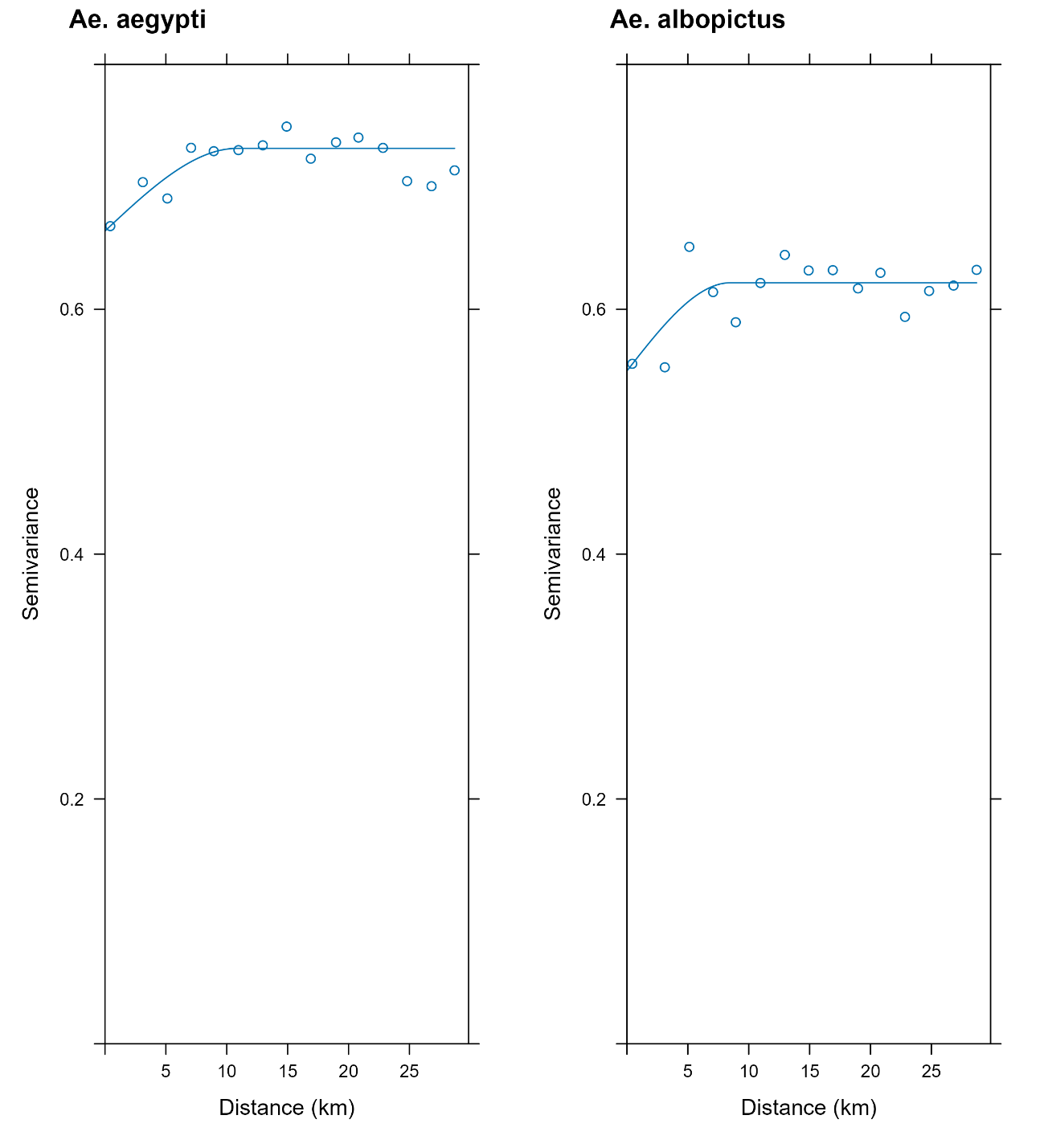


**Supplemental Figure 6.** Semivariograms for *Ae. aegypti* and *Ae. albopictus*, respectively. Range values for semivariogram sills (i.e., when spatial dependence of species abundance is no longer increasing with the distance between points) were 13km (sill = 0.0613) and 9km (sill = 0.0747), respectively.

**Supplemental Table 1.**

**Table 1.**

| Full Name | Description |
| --- | --- |
| Population Density | —- |
| Impervious Surface | —- |
| Population Size | —- |
| Development_1/2/3/4 | Level of development |
| Deciduous/Grass | % of land that is classified as deciduous or grassland |
| House Age | Mean home age (2024-year home was built) |
| Mean Income | Mean income of individuals above 18 years old |
| Population Age | Mean population age |
| Mean House Value | Mean value of homes built |
| Poverty Percentage | % of Population within Tract that is below poverty line |
| **Cloud Cover** | Total cloud cover as an area fraction |
| **Mean Dew Point** | —- |
| **Temperature Mean/Min/Max** | Mean/Min/Max temperature |
| **Vapor Pressure Deficit Min/Max** | —- |
| **Precipitation** | Amount of precipitation |
| **Mean Wind Speed** | Wind speed at 10 meters above ground |
| **Atmospheric Pressure** | Atmospheric air pressure at mean sea level |
| **Soil Moisture**^+; ++^ | Soil moisture (0-7 cm above ground) |
| **Soil Temperature^+; ++^** | Soil temperature (0-7 cm above ground) |
| Park Density | Density of parks (playgrounds, parks) |
| Entertainment Services Density**^+; ++^** | Density of entertainment services (theaters, cinema, community center, events & music venue, social center) |
| Shopping Centers Density**^+; ++^** | Density of shops (malls, brick and mortar, etc.) |
| Public Services Density**^+; ++^** | Density of schools (and libraries) |
| Road Density | Density of (residential) roads |

**Table 1.** Full list of variables extracted for the purposes of predicting *Ae. aegypti* and *Ae. albopictus* counts and their API source. Bold indicates that one- and two-week lags were also included.

+ - Excluded from the *Ae. aegypti* model after feature selection.

++ - Excluded from the *Ae. albopictus* model after feature selection.

**Supplemental Table 2.**

**Table 2.**

| *Model* | R^2^ | RMSE |
| --- | --- | --- |
| *Ae. aegypti RF (out of box)* | 37.29 | 0.647 (0.630 - 0.665) |
| *Ae. albopictus RF (out of box)* | 30.33 | 0.654 (0.636 - 0.673) |
| *Ae. aegypti RF (tuned)* | 37.52 | 0.649 (0.632 - 0.667) |
| *Ae. albopictus RF (tuned)* | 30.59 | 0.652 (0.634 - 0.671) |

**Supplemental Table 2**. Random Forest regression model with default parameters and tuned performance values.

**Supplemental Table 3**

| **Season** | ***Co-occurrence frequency (%)*** |
| --- | --- |
| December-February | 8.70 |
| March-May | 34.60 |
| June-August | 30.53 |
| September-November | 25.00 |

**Supplemental Table 3.** Observed co-occurrence frequency between *Ae. aegypti* and *Ae. albopictus* segmented by season.

**Supplemental Table 4.**

| Year | *Ae. aegypti* | *Ae. albopictus* |
| --- | --- | --- |
| 2015 | 0.715 | 0.751 |
| 2016 | 0.783 | 0.554 |
| 2017 | 0.685 | 0.618 |
| 2018 | 0.971 | 0.573 |
| 2019 | 0.503 | 0.730 |
| 2020 | 0.702 | 0.647 |
| 2021 | 0.694 | 1.07 |
| 2022 | 1.16 | 0.623 |

**Supplemental Table 4.** Mean abundance of log(abundance + 1) scaled counts, segmented by year for *Ae. aegypti* and *Ae. albopictus*.
